# Cannabis Policy Changes and Adolescent Cannabis Use: Evidence from Europe

**DOI:** 10.3390/ijerph18105174

**Published:** 2021-05-13

**Authors:** Elisa Benedetti, Giuliano Resce, Paolo Brunori, Sabrina Molinaro

**Affiliations:** 1 Italian National Research Council, Institute of Clinical Physiology (CNR-IFC), via Moruzzi, 1, 56124 Pisa, Italy; elisa.benedetti@ifc.cnr.it; 2 Centro Studi SOSE S.p.A. (Ministry of Economy and Finance), via Mentore Maggini, 48C, 00143 Rome, Italy; giuliano.resc@gmail.com; 3 Department of Economics and Management, University of Florence, via delle Pandette, 32, 50127 Florence, Italy; paolo.brunori@unifi.it

**Keywords:** drug policy, cannabis use and availability, adolescents, ESPAD, Differences-in-Differences

## Abstract

Cannabis accounts for the largest share of the illicit drug market, with a high prevalence of use even among adolescents. To tackle this longstanding problem, many kinds of reforms to national cannabis control policies have been implemented in Europe, but their effectiveness is still unclear. This paper analyses the association between selected categories of cannabis policy reforms and changes in perceived cannabis availability and patterns of use among adolescents. Data from 20 European countries across 15 years were drawn from a novel database of the European school Survey Project on Alcohol and other Drugs (ESPAD). Our analysis is based on a Difference-in-Differences design, which application is allowed by the fact that only thirteen out of the twenty countries included implemented policy changes. The results suggest that selected categories of reforms influence the availability and prevalence of cannabis use. In particular, some forms of restrictive intervention reduce the general prevalence of use and more liberal reforms seem linked to an increase in the share of students initiating use of cannabis. We find no evidence of an effect of policy changes on the share of frequent users, which are presumably those more likely to develop use-related health consequences.

## 1. Introduction

Cannabis policy is a topic of constant discussion and changes worldwide. This is because, notwithstanding the coordinated efforts to disrupt cannabis market, both supply and consumption indicators have constantly increased over the past decades [1]. It is estimated that in Europe around 15% of young adults (aged 15–34) used cannabis in 2019, and the prevalence reaches 19% when only 15- to 24-year-olds are considered [2].

Since 2013, Uruguay, 10 jurisdictions in the United States and Canada have passed laws that license the production and retail sale of cannabis to adults for non-medical purposes, often referred to as recreational use.

In parallel, a renewed debate about reforms to the national cannabis policies has developed in Europe [3,4]. In fact, although there is some European Union regulation concerning cannabis trafficking offences, legislative responses to unauthorised cannabis use and minor possession are still primarily responsibility of individual member states and therefore little harmonised [5,6]. As an example, cannabis policies range from the more liberal example of the Netherlands, with a system of limited distribution, to countries like Hungary, where personal possession of cannabis is punishable with imprisonment. Furthermore, some countries legally treat cannabis like other drugs, whilst in others penalties for cannabis are lower, typically because the level of harm that the use of the drug may cause is taken into consideration (Pacula et al., 2005). As an outcome, over the past years several European countries have implemented policy reforms modifying the size of the penalties for cannabis possession for personal use: despite a general trend to reduce punishments, few countries moved in the opposite direction. Some countries have reduced penalties for low-level offences, have removed criminal sanctions for possession or use, or have introduced formal or informal procedures that decrease the likelihood of sanctions being applied [4]. Others have increased penalties for personal possession, either treating them as criminal or administrative offences [7]. This results in a variety of policy approaches running in parallel in Europe, which range from administrative to criminal offences for personal cannabis possession [8], with the notable exception of the Dutch system.

The potential effect of policy reforms to the treatment of cannabis possession for recreational use on rates of cannabis use is a topic of considerable debate [4,9,10]. However, empirical research on the effects of the different types of control policies is still limited [11,12]. Gathering scientific evidence firstly on whether and which type of cannabis policy reforms are able to affect the availability of the substance and the prevalence of use, and secondly by which type of users and by how much seems crucial in order to understand their public health impacts [12,13].

In particular, while cannabis policy changes are currently limited to adults, increasing attention is being devoted to the effects that these might have on adolescents [14,15,16]. This is because cannabis is by far the most popular illicit substance among youth, particularly in Europe, where adolescents report high rates of easy access to the substance [17] and show higher prevalence of cannabis use compared to the adult population [18]. Furthermore, research shows that initiation into cannabis use typically occurs during the mid to late teens [13,19] and that there is a strong positive relationship between early first use and the length and intensity of cannabis consumption during adulthood [20,21,22], with a range of possible associated poorer outcomes later in life [23]. In general, policies ruling cannabis-related offences are primarily targeted at adults and some authors suggest that they do not affect adolescent consumption [24]. Despite this, several authors suggest that policy changes might indirectly affect adolescents by modifying their access to cannabis and by contributing to shape the social norms of a society [9,13,25]. Most of the existing studies on cannabis use associated with cannabis control policy reforms has been conducted in the United States, Australia and Canada, and mainly focused on the adult population. The most recent studies analysing the possible effects of drug policies on youth participation have investigated two specific types of policy changes, i.e., legalisation of cannabis for recreational purposes [26,27,28,29,30,31] and legalisation of medical cannabis [16,30]. Findings are mixed, and overall they suggest that the passage of the laws did not relevantly affect youth.

In Europe, due to the scarcity of comparable data, very little work has been performed, and mainly focused on a single-country perspective [19,22,32,33]. To the best of our knowledge, only one study examined the associations between country-level cannabis control policies and cannabis use in the adolescent population including many European countries [34]. Although results suggested that liberalisation policies in general were associated with higher odds of some measures of adolescent cannabis use, a later study conducted on the same data did not confirm this result [35].

Despite the scarcity of previous studies, Europe constitutes an interesting case for conducting this type of research, particularly because the cannabis law reforms passed over the last 20 years in many countries generated significant variations in the intensity and trajectory of policy changes (towards a decrease or decrease of penalties), which offer and optimal ground for research [7,36]. Although data to assess the implementation and evolution of policies “on the ground” are limited, recent studies have highlighted the importance of paying attention to variability in specific policy provisions when trying to evaluate their effects, instead of using simple categorisations, for example binary variables to classify legalisation and non-legalisation [12]. Although challenging, the European case offers an optimal setting for this exercise.

In this study, we examine the association between changes in cannabis control policies and changes in adolescent perceived availability and self-reported use of cannabis in 20 European countries (13 countries where laws were changed and 7 countries which served as a control group) over a period of more than 15 years (1999–2015). Specifically, we reviewed existing literature [7,37] to characterize the types of cannabis control policy changes in each country, and applied a Differences-in-Differences (DiD) model to a novel database of the European school Survey on Alcohol and other Drugs (ESPAD). The DiD is a popular statistical technique that attempts to mimic an experimental research design using observational study data. It allows to find the effects of an intervention on specific outcomes, by comparing the differences in outcomes after and before the intervention between treated and untreated groups of units. In our context, *DiD* is applied to statistically assess the association between types of cannabis policy changes (considered as treatments) and cannabis perceived availability and use among adolescents (our outcome measures).

The main contribution of this paper is to address the scarcity of an European perspective in the study of the links between cannabis policy reforms and cannabis-related outcomes among adolescent. We aim to do so by going beyond a simple categorisation of policy changes and try to capture their variability. Also, to better investigate their links with adolescent perceived availability and use, we take into account different patterns of cannabis use. In fact, as for adults, also when focusing on teenagers, it is important to acknowledge that cannabis market is segmented into a number of different types of consumers and that they might react to the same policy in different ways [38].

## 2. Materials and Methods

### 2.1. Data

Data from the European school Survey Project on Alcohol and other Drugs (ESPAD) were used in this study. ESPAD is a repeated cross-sectional multinational survey conducted every four years since 1995, designed to provide nationally representative and comparable data on substance use and other risk behaviours among 16 years-old students in Europe [17].

In this survey, a cluster sampling design is used to sample the students who turn 16 years of age in the given survey year. In the majority of countries, class is the last unit in a multistage stratified sampling procedure. Participating countries adhere to common research guidelines to guarantee consistency in sampling, questionnaires, and survey implementation, and conform to the respective national ethics and data protection regulations. A standardised anonymous questionnaire is voluntarily completed in the classroom setting with paper-and-pencil or computer-assisted format. Sampling frame coverage, school, class and student participation rates were generally high in the considered period. Detailed information about survey representativeness, data collection methodology, and country participation rates in each survey year are reported in the dedicated reports [39,40,41,42,43].

For the present analysis, starting from the individual level data about 306,693 students from 20 countries collected in five ESPAD data collection waves (1999, 2003, 2007, 2011 and 2015), annual prevalences were calculated for each country for the set of variables referring to cannabis use and perceived availability, obtaining a balanced panel covering 20 countries in the interval 1999–2015 (a total of five observations per country). Table 1 provides an overview of the initial sample by country and year.

### 2.2. Cannabis Perceived Availability and Use

The first outcome of interest was the perceived availability of cannabis. In ESPAD, respondents are asked: “How difficult do you think it would be for you to get marijuana or hashish (cannabis) if you wanted?”. This question is asked to every student answering the questionnaire, both those who reported having ever used cannabis and those who did not. Response options included “Impossible", “Very difficult”, “Fairly difficult”, “Fairly easy”, “Very easy”, “Don’t know”. Responses “Fairly easy” and “Very easy” were merged to indicate perceived easy availability. This outcome was analysed among all students (independently of their use of cannabis), among non-frequent users and among frequent users defined as follows.

Reported use of cannabis was the second outcome of interest. In ESPAD it is provided by responses to the question: “On how many occasions (if any) have you used marijuana or hashish (cannabis)?”. This question is asked with reference to three different timeframes: in the lifetime, in the last 12 months and in the last 30 days. The answer options for all timeframes are: “0”, “1–2”, “3–5”, “6–9”, “10–19”, “20–39” and “40 or more”. Using the information regarding cannabis use in the last 12 months and in the last 30 days, individuals were classified into different types of users: all users (at least once in the last 12 months); experimenters (only 1–2 times in the last 12 months); non-frequent users (all users in the last 12 months, excluding those having reported use in the last 30 days with a frequency equal to or higher than 20–39 times); frequent users (having reported use in the last 30 days with a frequency equal to or higher than 20–39 times). We also conducted a sensitivity analysis focusing on the main finding of this paper, which is that the influence of the different types of cannabis policy changes on the prevalence of adolescent cannabis use is strongly related to the frequency of use. To this end, in Section 3.3 we re-estimate the models using as outcome the lifetime consumption of cannabis, instead of the consumption in the last year, for all users (at least once in the lifetime) and experimenters (only 1–2 times in the lifetime).

### 2.3. Country-Level Characteristics

Estimates on country-level GDP per capita in each survey year were obtained from the World Bank [44]. Per-capita GDP is used to proxy for the country socio-economic conditions, which have been associated with youth consumption of psychoactive substances [34,45,46]. Estimates on the country-level share of urban population in each survey year were calculated using the World Bank population estimates [44] and urban ratios from the United Nations World Urbanization Prospects [47]. The share of urban population is used as a proxy for the level of urbanisation of a country, which has been identified as a risk factor for substance misuse [48].

### 2.4. Classification of Cannabis Policy Changes in Europe

To characterise country-level cannabis control policy changes, we primarily relied on Pacula et al. [49] and Room et al. [37]. On this basis, a distinction is made between decriminalisation (change in the the status of cannabis use from a criminal to a non-criminal offence), depenalisation (reduction of the severity of the penalties) and increase of the penalties (either civil or criminal). Going specifically into the European case, following the European Monitoring Centre for Drugs and Drug Addiction (EMCDDA) [7], this distinction was further refined to perform the analysis: decriminalisation through reforms that remove the prison sentences for minor offences (RPSMO); depenalisation, where the offence is still criminal, but a reduction of the maximum prison sentence is operated (RMPS); depenalisation where the offence is still criminal but the likelihood of sanctions being applied is reduced by facilitating the closure of minor cases (FCMC); increase of the penalties, where the possession for personal use is a civil offence but the reform increases the penalties attached to it (INPP); increase of the penalties, where the possession for personal use is a criminal offence and the reform increases the penalties attached to it (IPP).

To better understand how the European countries included in our study have been categorised and understand each specific policy change, Table 2 provides an overview of the broad category into which the policy changes fall, the specific type of reform and a description of the situation pre- and post-reform in each country.

Seven countries that did not pass any cannabis law in the observed period were used in the analysis as a control group: France, Iceland, Latvia, Malta, the Netherlands, Norway, Sweden.

Four additional countries that reformed their cannabis control policies in the observed period were excluded from the analysis because the related ESPAD data were not available for all the considered years: the United Kingdom (2004), Estonia (2002), Luxembourg (2001) and Belgium (2003).

### 2.5. Statistical Analysis

To investigate the relationship linking the policy changes and the perceived availability and use of cannabis among adolescents, we use a Differences-in-Differences (DiD) approach. The basic strategy of DiD is to compare the difference in outcomes after and before a specific intervention between treated and untreated groups of units [50]. The *DiD* framework can also be used in a more general setting, where more than two time periods and more than two groups are considered [51,52]. In this paper we are interested in the effect of five different categories of cannabis policy changes (our interventions), as described in Table 2. On the basis of the generalized DiD [52], we estimate the effect of all the considered policies in the same regression, which is given by the following equation:

(1) $$Y_{st}=\theta_{s}+\lambda_{t}+\beta_{1}RPSMO_{st}+\beta_{2}RMPS_{st}+\beta_{3}FCMC_{st}+\beta_{4}INPP_{st}+\beta_{5}IPP_{st}+\beta_{6}X_{st}+\varepsilon_{st}$$

where:

- $\theta_{s}$ are country-level fixed effects;
- $\lambda_{t}$ are time-level fixed effects;
- $RPSMO_{st}=1$ in countries and years Removing the prison sentences for minor offences;
- $RMPS_{st}=1$ in countries and years Reducing the maximum prison sentence;
- $FCMC_{st}=1$ in countries and years Facilitating closure of minor cases;
- $INPP_{st}=1$ in countries and years Increasing the non-prison penalty;
- $IPP_{st}=1$ in countries and years Increasing the prison penalty;
- $X_{st}$ is the vector control variables containing country level per-capita GDP and share of urban population.

We estimate different models for the following outcomes ($Y_{st}$), as described in Section 2.2: share of students reporting easy access to cannabis, share of non-frequent users reporting easy access to cannabis, share of frequent users reporting easy access to cannabis; prevalence of all cannabis users (any use in the past year); prevalence of experimental users (only 1–2 times in the past year); prevalence of non-frequent users (any use in the past year, but less than 20 times in the past month); prevalence of frequent users (20 times or more in the past month).

Estimated $\beta_{i}$, for each one of the five policy categories (i=1,…,5), is the difference between the average outcome observed in the treated countries after the implementation of the reforms, and the average outcome that would be expected for the same countries, in the same time periods, given their level of GDP per capita and level of urbanisation. For Italy and Greece, in which the analysed reforms remained into force for a limited period, the dummy variable corresponding to the reform assumes value equal to one only in those years. These parameters can be interpreted as the possible effect of the reforms on the outcome of interest under the assumption of equal trends. This means that we are assuming that, in the absence of policy reforms, trends in prevalences of cannabis availability and use would have moved in tandem in treated and untreated groups of countries.

Two robustness checks were also conducted. First, we performed a sensitivity analysis by re-estimating the model using as outcome the use of cannabis in the lifetime instead of the use in the last 12 months. Second, we used a test of trend [50,53], which is also suitable for multivalued treatments and several groups, to validate the parallel trend assumption that trends in country prevalence of cannabis availability and use were not already different prior to passage of the cannabis reforms. The idea is to include the policy dummy for the pre-policy period. Adapting this test to our case study, we have augmented all the policies showing a significant effect by one lead (which in our case corresponds to a four-year interval). This way, if the trends in outcomes between treatment and control groups were the same prior to passage of the cannabis reforms, then the coefficient associated to those dummies should be not significant.

## 3. Results

Table 3 presents the descriptive statistics of our outcome variables. Overall, the perceived easy availability of cannabis showed an average constant path across the years, while the use of cannabis slightly increase in the 1999–2015 interval. In particular, the frequent use of cannabis involved on average the 0.41 percent of students in 1999 and the 0.68 percent in 2015.

### 3.1. Policy Changes and Perceived Availability

Table 4 presents the DiD estimates of the effect of the five categories of cannabis policy changes on three different outcomes: the share of students reporting easy access to cannabis, the share of non-frequent users reporting easy access to cannabis, and the share of frequent users reporting easy access to cannabis.

The results reported in the first column of Table 4 show that only one category of policy reforms is associated to a significant change in the share of students reporting easy access to cannabis: in those countries which increased the non-prison penalties (INPP), the reform is associated to a significant decrease (5.8 percentage points), while in all other countries no significant effect is detected.

The model reported in the second column shows similar results: only in those countries implementing INPP, the reform is associated to a decrease of 5.7 percentage points in the share of non-frequent users reporting easy access to cannabis.

Finally, results in the third column show that no policy reform is associated to a significant change in the share of frequent users reporting easy access to cannabis.

### 3.2. Policy Changes and Cannabis Use

In this sub-section we check the hypothesis that cannabis policy reforms might have different effects on different user groups. To this end, Table 5 reports the DiD estimates on the following outcomes: prevalence of all cannabis users (any use in the past year); prevalence of experimental users (only 1–2 times in the past year); prevalence of non-frequent users (any use in the past year, but less than 20 times in the past month); prevalence of frequent users (20 times or more in the past month).

Results in the first column show that only two types of policy change are associated to significant changes in the prevalence of any cannabis use. Specifically, in the country implementing depenalisation through the facilitation of the closure of minor cases (FCMC), the policy change is associated to a significant increase in the share of cannabis users (6.6 percentage points), whilst in those countries increasing the non-prison penalty (INPP) the policy change is associated to a significant decrease (3.3 percentage points). The model for experimental users generally confirms the results of the first model but, in addition, a significant increase is observed in those countries implementing a reduction of the maximum prison sentence for cannabis possession (RMPS). This additional association is not confirmed in the model for non-frequent cannabis users (third column). Significant associations, similar to those for all users, are instead found in countries enacting policy reforms of the FCMC and INPP categories.

Interestingly, results in the fourth column show that when the analysis focuses on the prevalence of frequent users, no type of policy change shows a significant effect.

### 3.3. Robustness Checks

#### 3.3.1. Sensitivity Analysis

To perform this analysis we focus on the main finding of this paper, which is that the influence of the different types of cannabis policy changes on the prevalence of adolescent cannabis use is strongly related to the frequency of use. As illustrated in Section 2.5, in order to check the robustness of this finding we re-estimate the models presented in Table 5 using as outcomes the prevalence of all users and the prevalence of experimental users in the lifetime instead of in the past year. As shown in Table 6, results are in line with the main findings illustrated in Table 5. In particular, the policies increasing the non-prison penalty (INPP) have a significant negative effect, whilst the one facilitating the closure of minor cases (FCMF) has a significant positive effect. Also the estimates concerning the share of experimental users in the lifetime are in line with our main results: in the group of countries that reduced the maximum prison sentence (RMPS) and in the country that facilitated the closure of minor cases (FCMC) a significant increase is observed, whilst in those countries that increased the non-prison penalties (INPP) we observe a significant decrease.

#### 3.3.2. Parallel Trend Test

In Table 7 we present the results of the parallel trend test. As described in Section 2.5, to perform this test the policy reforms showing a significant association with changes in the perceived availability of cannabis and with changes in the past-year prevalence of cannabis use have been anticipated by four years. For convenience, we show the results for the following three main outcomes: share of students perceiving cannabis as easy to obtain; past-year prevalence of cannabis use and of experimental use. The coefficients associated to the leaded policy dummies are all non-significant, indicating that trends in outcomes were not already differentially changing in countries that enacted policy reforms and those that did not, prior to passage of the laws.

## 4. Discussion

This paper assessed whether the cannabis policy changes occurred in 13 European countries in the period 2001–2014 were associated with significant outcomes among adolescent students. In particular, to inform discussions about the evaluation of policy developments related to cannabis that might increase the availability of this substance within Europe, we analysed changes in the perceived availability of cannabis. In order to check the possible association with changes in the prevalence of use of this substance among adolescent students, we differentiated between different patterns of use (experimental, non-frequent and frequent users). This has been done in order to account for the fact that users are not equal, and that there is a group that, although restricted, is more at risk of developing cannabis-related problems, i.e. frequent users [23,54,55,56].

This study contributes to clarify the scarce and inconstant literature on European states [34,35], providing important information about policy outcomes and efficacy. Moreover, differently from previous studies that simply categorised cannabis control policies into a dichotomous measure (whether or not liberalised) [34], this study takes account of the fact that there is a great diversity of forms that relaxation or increase of prohibition can take in practice [25], by refining the investigation following the analysis proposed by EMCDDA [7]. In fact, ignoring the significant heterogeneity in these policies, has been highlighted to contribute to what appear to be mixed results from evaluations [57]. The categorisation used identified five main types of policy reforms on the basis of the treatment of cannabis possession for personal use. In order to better interpret the results, for each country a description of the situation pre- and post-reform has been provided. By combining data from five waves of the ESPAD survey, our data include a timespan of more than 15 years, covering the period before and after the implementation of each of the national drug policy reforms. Results are based on a DiD model.

Regarding the availability of cannabis, we find that none of the decriminalisation and depenalisation reforms seem to be linked to an increase in the perception of easy access to this substance by the general population of students, nor it is so among non-frequent and frequent users. This finding suggests that the common assumption that cannabis availability will increase with the relaxation of prohibition [2,58,59] might not apply to the European cases. This means that in a country like Portugal, where the personal possession of cannabis was decriminalised in 2001, the perceived availability of cannabis did not increase as a result of the reform compared to a country like France, where the possibility of incarceration for the possession of cannabis for personal use is still foreseen.

Among the policy reforms increasing the penalties for cannabis personal possession, those increasing the administrative penalties attached to this offence (INPP) are actually associated to a decrease in the rate of students perceiving cannabis as easy to obtain. This result might be a good indication as it has been demonstrated that those who believe they have easy access to cannabis have also a greater risk for uptake, higher consumption frequency, as well as the progression to regular use and abuse [60,61].

However, the fact that among users this association persists only for the non-frequent ones, suggests that the channels of access to the substance by frequent users, such as domestic production and supply networks [61], might not have undergone significant modifications. Since to our knowledge cannabis policies and changes in the perceived availability by adolescents were not previously explored in the European context, these results offer an interesting insight into the aforementioned relationship.

Concerning cannabis use outcomes, our results show that only some cannabis policy reforms were associated to significant changes in the prevalence. This can be considered an important finding in itself as it confirms that there is not an automatic link between cannabis policy and use [37], and that other factors may play an important role. Among these, we can mention information and prevention programs, but also the actual level of implementation and enforcement of reforms [57]. They can affect the perception of risk and knowledge of adolescents, as well the social acceptability of drug use in a country, which are in turn associated with substance use [30,58,61,62].

As highlighted by a previous study conducted in Europe [34], the heterogeneity found in the effects of cannabis policy reforms concerning the prevalence of use highlights the importance of making distinctions between different types of cannabis users. In fact, in line with previous findings [26,30] different results are obtained for different types of consumers. When considering all types of users, two categories of policies show an effect: among the more restrictive ones, only the one increasing the non-prison penalties (INPP) seem to significantly reduce overall cannabis use, and among the more liberal interventions, only the one favouring the discontinuation of criminal proceedings for minor cases (FCMC) is linked to an increase. These results are confirmed when focusing on experimental users or excluding frequent users from the analysis. Furthermore, those reforms reducing the maximum prison penalty for cannabis possession (RMPS) show a positive effect on the share of experimental users only. When finally considering only frequent users, i.e. students smoking cannabis daily or almost daily, the policy effects observed before disappear and no reforms seem to have an effect. This result is not in line with the finding from Shi et al. [34] indicating that cannabis liberalisation in Europe was associated with higher likelihood of regular use. It is instead supported by a revision of the same study that highlighted how, by implementing some statistical improvements, this association becomes statistically non-significant [35]. The finding is also in line with some recent within-country studies conducted to analyse some form of cannabis liberalisation policies in the US [63,64,65,66]. In these studies, no discernible pattern was detected suggesting an increases in cannabis frequent use among adolescent related to the legalisation of medical marijuana.

Overall, these results offer three main insights. First, the fact that some of the reforms reducing the penalties for cannabis possession are associated to an increase in some measures of adolescent cannabis use signal that those reforms might have somehow reduced stigma and perceptions of risk associated with cannabis use [13,67,68]. This is in light of the fact that no increase was observed concerning adolescent perceived easy access to the substance, indicating the the other main factor on which the policy reforms might have acted [13,16] did not change significantly. A shift in social norms regarding cannabis use may have, instead, increased cannabis use among experimental (RMPS and FCMC) and non-frequent users (only FCMC). On the contrary, in those countries where the civil penalties for cannabis possession where increased (INPP), the reduction in the share of experimental and non-frequent users was coupled by a reduction in their perception of cannabis availability. This might indicate that this kind of policy was effective in reducing access through informal channels [16,61] or increasing the price of this substance on the black market [16,69] for those sub-populations of users.

Second, it has to be considered that one fundamental reason for increasing the level of prohibition is that positive social externalities should be larger than the social costs of repression and private benefits for users [69,70]. In fact, the failure in achieving this objective has lead several countries to move towards depenalisation and legalisation in recent years [4]. Our results confirm that some of these reforms (INPP) are linked to a reduction in the share of students approaching this substance, which is in line with this objective. However, the fact that no reduction is observed in the share of frequent users, who are those at higher risk, signal a limited public health impact of this approach among adolescents at higher risk, which might in turn reduce its social externalities. A similar reasoning can be applied to the Hungarian case (IPP), which applies the more severe punishments for cannabis possession, and where no significant change was observed either in the perceived availability or in the share of cannabis users following the policy reform.

Finally, the absence of a significant decrease in the share of frequent cannabis users associated to any of the policy changes, might signal a limited role of policies overall in achieving results among this high-risk population. In this light, investments in evidence-based adolescent substance use prevention programs would be advisable [16,71,72]. Given the fact that we did not find any significant decrease in the perceived accessibility to cannabis for frequent users, an interesting perspective would be to focus on resilence factors, which may increase the unwillingness to use the drug, even when drug use opportunities are available [58].

## 5. Limitations

This study has some limitations, some of which are common to other studies using cross-sectional data, that we aim to address in future research. First, since our estimates rely on self-reported survey data, there might be the concern that changes in drug policy influence the way individuals answer the survey. For example, if with more liberal policies and less severe punishments in place people were more prone to admit drug use when asked about it in a survey. Although issues of truthfulness are more likely to arise when surveys are administered by personal interview, whilst in our case the ESPAD survey is anonymous and self-administered respecting privacy conditions, we cannot completely rule out this hypothesis. This is one of the trade-offs that research on socially undesirable and illegal behaviours is confronted with. Second, our analysis is based on country-level prevalence measures, which do not account for individual-level factors that may confound the relationship between cannabis policy reforms and cannabis perceived availability and use among adolescents. This is because, while a number of individual-level variables are available in the last waves of the ESPAD study for many countries, they are not available for the whole time span and countries considered. Confronted with this trade-off, consistently with our research question we opted for maintaining in the analysis many countries and the largest time span possible to be able to provide an European picture. Finally, this research, as most part of the previous studies on the topic, does not include an analysis of the actual level of enforcement of the policy reforms, nor of social approval of cannabis use, due to data limitation. This is something that would be important to explore in the future, when some more information about these aspects will possibly be available.

## 6. Conclusions

Changes to cannabis control policies are a topic of constant public debate. So far research on their relationship with changes in adolescent cannabis use has been challenged by the scarcity of available data and did not reach conclusive evidence. Despite its limitations, this study for the first time examined the association between changes in policies ruling cannabis possession in Europe and cannabis perceived availability and use in the adolescent population, a particularly vulnerable group for drug initiation. Our study showed to some forms of liberalisation were associated to an increase in some measures of cannabis use, whilst some reforms increasing the penalties were associated to a decrease both in the perceived availability of the substance and in some measures of cannabis use. However, no policy change was associated to a decrease in the frequent use of cannabis nor in the perception of availability of this substance by frequent users. This suggests that any cannabis control policy should be accompanied by investments in evidence-based adolescent substance use prevention programs. To be effective, these might possibly target resilience factors in order to increase the unwillingness to use the drug, even when actual opportunities to do so are available.

## Figures and Tables

**Table 1:** Sample size by country and year.

	1999	2003	2007	2011	2015	Total
Croatia	3454	2823	2947	2953	2490	14,667
Czech Rep.	3478	3078	3805	3826	2689	16,876
Denmark	1497	2442	844	2105	1624	8512
Finland	2945	3182	4902	3692	3960	18,681
France	2177	2090	2843	2463	2641	12,214
Greece	2160	1871	2990	5654	3168	15,843
Hungary	2669	3037	2758	2995	2692	14,151
Iceland	3342	1503	3421	3242	2604	14,112
Italy	4041	4693	9396	4657	3878	26,665
Latvia	2238	2782	2231	2542	1059	10,852
Malta	3593	3363	3601	3307	3171	17,035
Netherlands	2613	2019	2055	2030	1680	10,397
Norway	3582	3631	3077	2684	2320	15,294
Poland	3208	5770	2080	5818	11,645	28,521
Portugal	3496	2827	3049	1889	3355	14,616
Romania	2304	4214	2224	2678	3327	14,747
Slovak Rep.	2402	2098	2390	1902	2179	10,971
Slovenia	2304	2706	3037	3113	3390	14,550
Sweden	3243	3142	3078	2451	2485	14,399
Ukraine	2833	3998	2336	2132	2291	13,590
Total	57,579	61,269	63,064	62,133	62,648	306,693

**Table 2:** Classification of changes in cannabis law regarding possession for personal use occurred in Europe from 2001 to 2014.

Category	Country	Year	Before Change	After Change
RPSMO	Portugal	2001	Criminal offence punishable with up to one year’s imprisonment.	Decriminalised and offenders are referred to a commission deciding on the administrative sanction to apply (e.g., a fine).
	Slovenia	2005	Criminal offence punishable by up to 30 days imprisonment, 5 days for a small quantity of drug.	Decriminalised and is now punishable by a fine.
	Croatia	2013	Criminal offence punishable by up to 3 years’ imprisonment.	Decriminalised and in any amount is punished by a fine.
RMPS	Finland	2001	Criminal offence punishable by up to 2 years in prison.	The maximum penalty was lowered to 6 months in prison, allowing the prosecutor to deal with the majority of cases with a fine.
	Greece	2006–2013	Criminal offence punishable by up to 5 years in prison.	The maximum penalty was lowered to 1 year in prison (not entered in the criminal record).
	Czech Republic	2010	Personal possession of ‘‘greater than small’’ quantities of cannabis resulted in a jail sentence of up to 2 years.	Personal possession of cannabis for personal use is punishable by up to 1 year.
	Romania	2004	The penalty applied to the possession for personal use ranged from 2 to 5 years in prison, without distinction by drug.	Drugs were distinguished between high risk and risk categories: the penalty for cannabis (risk category) was lowered to 6 months’ to 2 years’ imprisonment.
	Slovak Republic	2005	Criminal offence punished by up to 3 years imprisonment.	Up to 3 doses punished by up to 3 years imprisonment, for a larger amount by up to 5 years (previously categorised as a trafficking offence).
FCMC	Poland	2011	Criminal offence punished by a maximum of 3 years imprisonment	The possession of drugs for personal use may now remain unpunished, subject to the discretion of the prosecutor/judge.
INPP	Denmark	2004	Criminal offence but did not result in prosecution, and was instead punished by a warning.	It remains a criminal offence: normal response for minor quantities is a fine, the size of which depends on type/quantity of the drug involved.
	Italy	2006–2014	Decriminalised and cannabis classified as a soft drug punishable with administrative sanctions. In 2006 the distinction between soft and hard drugs was eliminated. The administrative sanctions for soft drugs increased with hard drugs to a max. of 1 year.	In 2014, the Constitutional Court repealed the 2006 law and penalties for minor personal use offences were reinstalled to 1-3 months for cannabis and other less dangerous drugs.
	Ukraine	2010	Administrative offence if in the amount of a small size.	If the amount of drugs possessed does not exceed the ‘small’ amount, it remains an administrative offence, but the legal threshold of “small”, “large” quantities have been significantly reduced. Over the "small" threshold, a criminal case is opened.
IPP	Hungary	2013	Criminal offence, punished by up to two years imprisonment.	Punishment remains up to two years in prison if it involves small quantities, but other penalties are now one to five years for a basic offence, increasing significantly in certain circumstances.

Source: Authors’ elaboration on EMCDDA (2017a); RPSMO = Removal of the prison sentences for minor offences; RMPS = Reduction of the maximum prison sentence; FCMC = Facilitation of closure of minor cases; INPP = Increase of the non-prison penalty; IPP = Increase of the prison penalty.

**Table 3:** Descriptive statistics of outcome variables.

Prevalence (%)	N. Countries	1999 Mean *St.D.*	2003 Mean *St.D.*	2007 Mean *St.D.*	2011 Mean *St.D.*	2015 Mean *St.D.*
Perceived cannabis availability	20	30.42	32.15	33.07	31.34	30.38
		13.92	15.15	16.03	12.49	11.52
Past-year use of cannabis (any)	20	11.42	12.54	12.01	12.95	12.11
		6.40	8.44	8.54	6.77	7.05
Past-year experimental use of cannabis	20	5.00	5.63	5.50	6.26	5.68
		2.26	2.89	3.13	3.06	3.03
Frequent use of cannabis	20	0.41	0.60	0.63	0.67	0.68
		0.41	0.62	0.68	0.60	0.64
Lifetime use of cannabis (any)	20	15.07	17.08	15.99	16.82	15.65
		8.08	10.25	10.70	9.31	9.04
Lifetime experimental use of cannabis	20	6.36	7.10	6.84	7.48	6.58
		2.77	3.07	3.52	3.79	3.40

Notes: Author’s elaboration on data from the European School Survey Project on Alcohol and Other Drugs (ESPAD). Cannabis availability = percentage of students rating cannabis as either ‘fairly easy’ or ‘very easy’ to obtain; Past-year use of cannabis (any) = use of cannabis >= 1–2 times in the last 12 months; Past-year experimental use of cannabis = use of cannabis = 1–2 times in the last 12 months; Frequent use of cannabis = use of cannabis >= 20–29 times in the last 30 days; Lifetime use of cannabis (any) = use of cannabis >= 1–2 times in the lifetime; Lifetime experimental use of cannabis = use of cannabis = 1–2 times in the lifetime; Sample size: Average (country/year) = 3092.35 students, Min = 844 (Denmark 2007), Max = 11,645 (Poland 2015).

**Table 4:** Estimated association between cannabis policy reforms and changes in the share of all students, non-frequent users and frequent users considering easy to find cannabis.

	All Students		Non-Frequent Users		Frequent Users	
	Coeff.	S.E.	Coeff.	S.E.	Coeff.	S.E.
Perc. GDP	0.000	(0.000)	0.000	(0.000)	0.000	(0.000)
Urban Pop.	0.311	(0.412)	0.360	(0.402)	−0.050 *	(0.021)
RMPS	−2.158	(2.508)	−2.093	(2.448)	−0.065	(0.130)
RPSMO	−2.034	(3.199)	−2.134	(3.122)	0.100	(0.166)
FCMC	6.568	(4.954)	6.604	(4.835)	−0.036	(0.257)
INPP	−5.783 *	(2.891)	−5.675 *	(2.821)	−0.108	(0.150)
IPP	−1.855	(5.826)	−1.640	(5.685)	−0.036	(0.257)

Sample size: Average (20 countries/5 years) = 3092.35 students, Min = 844 (Denmark 2007), Max = 11,645 (Poland 2015); * *p* < 0.05.

**Table 5:** Estimated association between cannabis policy reforms and changes in past-year prevalence of all cannabis users, experimental, non-frequent and frequent users.

	All Users		Experimenters		Non-Frequent Users		Frequent Users	
	Coeff.	S.E.	Coeff.	S.E.	Coeff.	S.E.	Coeff.	S.E.
Perc. GDP	0.000	(0.000)	0.000	(0.000)	0.000	(0.000)	0.000	(0.000)
Urban Pop.	0.060	(0.208)	−0.011	(0.077)	0.079	(0.211)	−0.049 *	(0.022)
RMPS	0.497	(1.265)	0.994 *	(0.469)	0.554	(1.184)	−0.087	(0.136)
RPSMO	1.033	(1.614)	0.985	(0.599)	0.886	(1.503)	0.067	(0.173)
FCMC	6.607 *	(2.499)	2.849 **	(0.927)	6.588 **	(2.341)	−0.011	(0.268)
INPP	−3.333 *	(1.458)	−1.792 **	(0.541)	−3.239 *	(1.374)	−0.109	(0.156)
IPP	−0.959	(2.939)	−0.032	(1.090)	−0.588	(2.715)	−0.225	(0.315)

Sample size: Average (20 countries/5 years) = 3092.35 students, Min = 844 (Denmark 2007), Max = 11,645 (Poland 2015); * *p* < 0.05 ** *p* < 0.01.

**Table 6:** Estimated association between cannabis policy reforms and changes in lifetime prevalence of all cannabis users and experimental users.

	All Users		Experimenters	
	Coeff.	S.E.	Coeff.	S.E.
Perc. GDP	0.000	(0.000)	0.000	(0.000)
Urban Pop.	−0.065	(0.245)	0.039	(0.098)
RMPS	1.921	(1.494)	2.016 **	(0.597)
RPSMO	1.434	(1.906)	1.463.	(0.762)
FCMC	7.963 **	(2.951)	3.646 **	(1.179)
INPP	−5.443 **	(1.722)	−2.458 ***	(0.688)
IPP	−0.075	(3.470)	0.522	(1.387)

Sample size: Average (20 countries/5 years) = 3092.35 students, Min = 844 (Denmark 2007), Max = 11,645 (Poland 2015); . *p* < 0.1 ** *p* < 0.01 *** *p* < 0.001.

**Table 7:** Parallel trend test of estimated association between cannabis policy reforms and changes in perceived availability of cannabis by all students, past-year prevalence of all cannabis users and experimental users.

	Perceived Availability		All Users		Experimenters	
	Coeff.	S.E.	Coeff.	S.E.	Coeff.	S.E.
Perc. GDP	0.000	(0.001)	0.000	(0.000)	0.000	(0.000)
Urban Pop.	−0.005	(0.597)	0.079	(0.298)	−0.024	(0.109)
RMPS	−2.024	(3.024)	0.385	(1.483)	0.468	(0.574)
RPSMO	−4.902	(4.370)	−0.845	(2.137)	0.372	(0.772)
FCMC	7.484	(6.767)	6.780.	(3.978)	2.146	(1.432)
INPP	−6.875.	(4.027)	−1.721	(1.977)	−1.252.	(0.712)
lead(RMPS)					0.814	(0.661)
lead(FCMC)			−0.503	(3.418)	1.048	(1.234)
lead(INPP)	−1.733	(3.635)	−2.000	(1.772)	−0.533	(0.641)

Sample size: Average (20 countries/5 years) = 3092.35 students, Min = 844 (Denmark 2007), Max = 11,645 (Poland 2015); . *p* < 0.1.

## Data Availability

Restrictions apply to the availability of these data. Data were obtained from the ESPAD project and are available at URL http://www.espad.org/ upon application, accessed on 11 May 2021.

## Abbreviations

The following abbreviations are used in this manuscript:

RPSMO	Removal of the prison sentences for minor offences
RMPS	Reduction of the maximum prison sentence
FCMC	Facilitation of closure of minor cases
INPP	Increase of the non-prison penalty
IPP	Increase of the prison penalty
